# A gender-based investigation of risk factors for infectious complications after percutaneous nephrolithotomy for kidney stones: insight for personalized management

**DOI:** 10.1007/s00345-026-06275-7

**Published:** 2026-02-18

**Authors:** Federica Passarelli, Ludovico Maria Basadonna, Fabio Ciamarra, Gianpaolo Lucignani, Francesco Ripa, Stefano Paolo Zanetti, Elisa De Lorenzis, Giancarlo Albo, Emanuele Montanari, Luca Boeri

**Affiliations:** 1 Department of Urology, IRCCS Fondazione Ca’ Granda Ospedale Maggiore Policlinico, Via della Commenda 15, 20122 Milan, Italy; 2 University of Milan, Department of Clinical Sciences and Community Health Dipartimento di Eccellenza , Milan, Italy

**Keywords:** Gender, Risk factors, Sepsis, Percutaneous nephrolithotomy, Infectious complications, Stone

## Abstract

**Purpose:**

To investigate gender-related predictors of infectious complications after percutaneous nephrolithotomy (PCNL) in a large cohort of patients with kidney stones.

**Materials and methods:**

We retrospectively analysed data from 492 consecutive patients who under-went PCNL at a single tertiary-referral academic center (01/2016-09/2024). Patient’s demographics, stones characteristics and operative data were collected. Stone-free status was defined as no residual stones. Complications were graded according to modified Clavien classification. Descriptive statistics and logistic regression models were used to identify factors associated with postoperative infectious complications according to patient’s gender.

**Results:**

Females accounted for 39.2% of the population and showed a significantly higher rate of postoperative infectious complications compared to males (24.9% vs. 16.7%, p = 0.02). Preoperative positive bladder urine cultures were more frequent in females (27.9% vs. 16.1%, p = 0.01), as were infected stones (32.6% vs. 17.4%, p = 0.001). When stratified by gender, in males, infections were significantly associated with preoperative positive urine culture (p < 0.001) and residual stone status (p = 0.02), in females, longer operative time (p = 0.01) and residual stones (p = 0.02) were the main predictors. ROC curve analysis confirmed sex-specific thresholds for infection risk: in females, a stone volume ≥3.9 cm³ and operative time ≥96 minutes; in males, a stone volume ≥5.1 cm³ and operative time ≥137 minutes.

**Conclusion:**

Female patients had higher risk of infections post PCNL than men. Surgical factors are associated with infections complications in female, while a combination of procedural and patient’s factors were found in men. In females, infectious complications occurred at lower stone volume and shorter operative time, suggesting that a gender-based risk strategy should be performed to prevent infections after PCNL.

## Introduction

Percutaneous nephrolithotomy (PCNL) is the gold standard surgical treatment for large and complex kidney stones, offering high stone clearance rates, minimal surgical trauma, and rapid postoperative recovery [1, 2]. This approach is especially favored for challenging cases, including staghorn and infection-related stones [3, 4]. Despite its effectiveness, PCNL carries a risk of various postoperative complications, ranging from minor urinary leakage to severe bleeding and infectious events, which can significantly impact patient post-operative outcomes [5]. Among these, postoperative fever is the most frequent complication, occurring in 21.0% to 39.8% of cases [6]. Notably, fever is an established predictor of severe infectious outcomes, including sepsis and septic shock, highlighting its critical importance in patient management [6]. Furthermore, postoperative fever represents a key clinical component of the systemic inflammatory response syndrome (SIRS) and may reflect the early onset of a systemic inflammatory reaction even in the absence of positive microbiological cultures [7]. Several clinical and procedural factors have been identified as predictors of infectious complications following PCNL and are routinely considered in perioperative risk stratification. These include positive preoperative bladder urine culture (BUC), positive stone cultures, diabetes mellitus, multiple access tracts, larger stone burden, presence of hydronephrosis, staghorn calculi, struvite stone composition, residual fragments and prolonged operative time [8, 9]. 

In recent years, among various risk factors, growing attention has been directed toward gender-related differences in the epidemiology and clinical course of urolithiasis, particularly in relation to infectious complications. Recent studies have consistently shown that female patients exhibit a higher risk of developing stone-related infections and urosepsis compared to their male counterparts, a disparity potentially attributable to anatomical, hormonal, and microbiological differences in the urinary tract [10, 11]. 

Despite these observations, the impact of gender on infection-related outcomes has not been systematically investigated in the context of surgical interventions such as PCNL, leaving a critical gap in our understanding of sex-specific risk stratification and perioperative management. Most available data focus on established clinical or procedural risk factors in the general population, with limited attention paid to sex-specific variables that may influence infection risk after surgical stone treatment. This represents a critical gap in literature, particularly given the increasing emphasis on personalized medicine and stratified perioperative care. Therefore, the aim of our study was to investigate gender-related predictors of infectious complications following PCNL in a large, real-life cohort of patients with kidney stones.

## Materials and methods

We retrospectively analyzed data from 527 patients who underwent PCNL at a tertiary referral academic center between January 2016 and September 2024. Demographic and clinical data were collected. Health-relevant comorbidities were assessed using the Charlson Comorbidity Index (CCI) [12]. 

Stone characteristics were evaluated based on non-contrast-enhanced computed tomography (CT), which was performed in all patients prior to surgery. Stone volume was calculated using the ellipsoid formula (length × width × height × π × 1/6) [13]. The mean stone density in Hounsfield units (HU), presence of multiple stones and staghorn calculi were assessed. The results of preoperative bladder urine cultures were recorded [14]. Patients with negative results were administered a single dose of prophylactic parenteral antibiotics prior to surgery, with cephalosporins preferred in the absence of allergies. Patients with asymptomatic bacteriuria received targeted antimicrobial treatment for 48 to 72 h before surgery. If leukocytosis, urinary symptoms, or fever were present, the procedure was delayed until the patient completed a full course of antibiotics and obtained a sterile urine culture [15, 16]. Stones were classified as infected based on microbiological analysis.

All surgical procedures were performed under general anesthesia by experienced urologists using either standard or mini-PCNL (mPCNL) techniques. Among the mPCNL procedures, vacuum-assisted mPCNL (vamPCNL) [17, 18] and vacuum-cleaner mPCNL (vcmPCNL) [3] were adopted as previously described. The use of multiple percutaneous access tracts was recorded. Operative time was measured from the initial puncture to the completion of stone removal. Postoperative outcomes included length of hospital stay, presence of infectious complications and overall complication rate, classified according to the PCNL-adjusted Clavien-Dindo classification [19]. Infectious complications were defined as systemic inflammatory response syndrome (SIRS) with bacteremia or bacteriuria, as previously described [20]. Patients underwent a CT scan within three months postoperatively to assess the presence of residual fragments [21]. 

We excluded patients with congenital renal anomalies (*n* = 3), cases involving additional concomitant procedures other than mPCNL and endoscopic combined intrarenal surgery (*n* = 7), scheduled staged procedures for large stone burden (*N* = 27). A final sample of 492 (93.6%) patients was considered for statistical analysis.

Data collection followed the principles outlined in the Declaration of Helsinki. All patients signed an informed consent agreeing to share their own anonymous information for future studies. The study was approved by our Hospital Ethical Committee (Prot. 25508).

### Statistical analysis

Distribution of data was tested with the Shapiro–Wilk test. Data are presented as medians (interquartile range; IQR) or frequencies (proportions). Statistical analysis was conducted in several stages. Initially, descriptive statistics were used to characterize the entire study cohort. Subsequently, differences in clinical and operative parameters between patients who developed infectious complications and those who did not (-infections) were assessed using the Mann–Whitney U test and Chi-square test, as appropriate.

To identify potential predictors of postoperative infectious complications, across the entire cohort, both univariate and multivariate logistic regression analyses were performed. Following the identification of gender as a potential risk factor for infections, further analyses were conducted to evaluate clinical and laboratory differences between male and female patients using the Mann–Whitney U test and Fisher exact test.

Next, we examined whether clinical characteristics and postoperative infectious risk factors varied by gender. Descriptive statistics were used to compare clinical and surgical variables between +infections and -infections patients within each gender group. Univariate and multivariate logistic regression analyses were then applied separately within male and female subgroups to identify gender-specific risk factors for infectious complications.

Lastly, Receiver Operating Characteristic (ROC) curve analysis, along with the Youden index, was employed to determine optimal cutoff values for infections prediction in each gender group.

Statistical analyses were performed using SPSS v.28 (IBM Corp., Armonk, NY, USA). All tests were two sided and statistical significance level was set at *p* < 0.05.

## Results

Table 1 depicts demographic and clinical characteristics of the cohort, stratified by gender. In the whole cohort, median age and BMI were 58 years (IQR 49–66) and 24.7 kg/m² (IQR 22.2–28.1), respectively. Females accounted for 39.2% of patients, and 193 (39.2%) had a CCI ≥ 1. The median stone volume was 2.6 cm³ (1.1–5.1), with 72.9% having multiple stones and 32.9% staghorn stones. Preoperative positive BUC was present in 20.7% of patients, more frequently in females than males (27.9% vs. 16.1%, *p* = 0.01), and infected stones were also more common in females (32.6% vs. 17.4%, *p* = 0.001). mPCNL was performed in 416 cases (84.6%), with a median operative time of 95 min (IQR 75–130). Infectious complications occurred in 19.9% of patients. Of these, 5.9% developed sepsis and 0.6% required intensive care unit admission. Among patients who developed infectious complications, Escherichia coli was identified in 38% of cases, Proteus mirabilis in 21%, Enterococcus faecalis in 29%, and mixed microbial flora in 22%. Concordance between preoperative and postoperative cultures was observed in 44.8% of cases. Overall, 23.9% of complications were low-grade (Clavien-Dindo I–II) and 5.5% high-grade (IIIa/b). The overall stone-free rate (SFR) was 78.9%.

**Table 1:** Demographic characteristics of the study cohort according to gender (No. = 492)

	Total	Male	Female	P Value*
No. of patients [No. (%)]	492	299 (60.8)	193 (39.2)	
Age (years)				0.8
Median (IQR)	58 (49-66)	55 (48-64)	55 (44-62)	
Range	19 - 85	20 - 85	19 – 81	
BMI (kg/m^2^)				0.8
Median (IQR)	24.7 (22.2-28.1)	24.9 (22.6-27.8)	24.3 (21.2-28.7)	
Range	17.9 – 38.8	17.9 – 36.8	18.1 – 38.8	
CCI				0.9
Mean (SD)	0.7 (1.1)	0.8 (1.2)	0.4 (0.8)	
Median (IQR)	0 (0-1)	0 (0-2)	0 (0-1)	
Range	0 – 6	0 – 5	0 - 6	
CCI ≥1 [No. (%)]	193 (39.2)	118 (39.4)	75 (38.8)	0.8
Stone volume (cm^3^)				0.3
Median (IQR)	2.6 (1.1-5.1)	2.8 (1.0-5.6)	2.5 (1.2-4.5)	
Range	0.5 – 61.2	0.5 – 61.2	0.5 – 48.8	
Multiple stones [No. (%)]	359 (72.9)	224 (74.9)	135 (69.9)	0.2
Staghorn stone [No. (%)]	162 (32.9)	91 (30.4)	71 (36.8)	0.1
Mean stone density (HU)				0.6
Median (IQR)	848 (600-1000)	855 (607-1020)	831 (624-1000)	
Range	150 – 1983	150 – 1983	190 - 1500	
Preoperative positive BUC No. [(%)]	102 (20.7)	48 (16.1)	54 (27.9)	0.01
mPCNL procedure [No. (%)]	416 (84.6)	248 (82.9)	168 (87.0)	0.2
Preoperative indwelling stent [No.(%)]	51 (10.4)	32 (10.7)	19 (9.8)	0.6
Multiple access tracts [No. (%)]	78 (15.9)	47 (15.7)	31 (16.1)	0.8
Operative time (min)				0.1
Median (IQR)	95 (75-130)	110 (80-135)	105 (80-132)	
Range	30 – 180	40 – 180	30 - 180	
Hospitalization time (days)				0.5
Median (IQR)	5 (4-7)	5 (3-7)	4 (4-7)	
Range	2 – 50	2 – 50	2 – 42	
Infected stone [No. (%)]	115 (23.4)	52 (17.4)	63 (32.6)	0.001
Stone free rate [No. (%)]	388 (78.9)	240 (80.2)	148 (76.6)	0.2
Any complications [No. (%)]	145 (29.5)			
Postoperative complications [No. (%)]				
(Highest Clavien score)				
Clavien-Dindo I-II	118 (23.9)			
Clavien-Dindo IIIa/b	27 (5.5)			

*BMI: * body mass index; *CCI:* Charlson Comorbidity Index; *HU: * Hounsfield unit; *BUC*: Bladder urine culture; *mPCNL:* mini percutaneous nephrolithotomy

*P value according to the Mann-Whitney U test for continuous data and the Fisher Exact Test for categorical variables, as indicated

Table 2 reports demographic and clinical characteristics according to the occurrence of postoperative infectious complications. Infections were more frequent in females than males (24.9% vs. 16.7%, *p* = 0.02). Patients with infections had significantly larger stone volumes [5.3 (1.8–14.6) vs. 2.3 (0.9–4.2) cm³, *p* < 0.001], and a higher prevalence of multiple stones (*p* = 0.01) and staghorn calculi (*p* = 0.001). Preoperative positive BUC (*p* = 0.01) and infected stones (39.7% vs. 19.2%, *p* = 0.001) were also more common in the infected group. Patients with infections more often underwent standard PCNL (*p* = 0.01), required multiple access tracts (*p* = 0.01), had longer operative times [120 (90–156) vs. 90 (73–120) min, *p* < 0.001], and longer postoperative hospitalization (*p* < 0.001) than those without infections. SFR was significantly lower in patients with infections (61.2% vs. 83.2%, *p* = 0.001).

**Table 2:** Demographic characteristics of the study cohort according to the presence of sepsis (No. = 492)

	Infections	+ Infections	p value*
No. of patients [No. (%)]	394 (80.1)	98 (19.9)	
Age (years)			0.3
Median (IQR)	59 (49-67)	58 (49-66)	
Range	19 – 85	20 - 84	
Gender [No. (%)]			0.02
Female	145 (75.1)	48 (24.9)	
Male	249 (83.3)	50 (16.7)	
BMI (kg/m^2^)			0.5
Median (IQR)	24.7 (22.3-27.7)	25.0 (21.9-29.4)	
Range	17.9 – 38.8	18.1 – 37.9	
CCI			0.4
Mean (SD)	0.7 (1.1)	0.8 (1.2)	
Median (IQR)	0 (0-1)	0 (0-1)	
Range	0 – 6	0 - 5	
CCI ≥1 [No. (%)]	152 (38.5)	41 (41.8)	0.2
Stone volume (cm^3^)			<0.001
Median (IQR)	2.3 (0.9-4.2)	5.3 (1.8-14.6)	
Range	0.5 – 50.1	0.5 – 61.2	
Multiple stones [No. (%)]	276 (70.1)	83 (84.7)	0.01
Staghorn stone [No. (%)]	111 (28.2)	51 (52.0)	0.001
Mean stone density (Hounsfield unit)			0.2
Median (IQR)	850 (650-1050)	790 (595-930)	
Range	150 – 1983	300 - 1500	
Preoperative positive BUC No. [(%)]	66 (16.7)	36 (36.7)	0.01
Procedure type [No. (%)]			0.01
mPCNL	346 (87.8)	70 (71.4)	
standard PCNL	48 (12.1)	28 (28.5)	
Preoperative indwelling stent [No. (%)]	39 (9.8)	12 (12.2)	0.1
Multiple access tracts [No. (%)]	50 (12.7)	28 (28.6)	0.01
Operative time (min)			<0.001
Median (IQR)	90 (73-120)	120 (90-156)	
Range	30 – 180	48 - 180	
Hospitalization time (days)			<0.001
Median (IQR)	4 (3-6)	8 (5-12)	
Range	2 – 30	3 – 50	
Infected stone [No. (%)]	76 (19.2)	39 (39.7)	0.001
Stone free rate [No. (%)]	328 (83.2)	60 (61.2)	0.001

*BMI*: body mass index; *CCI:* Charlson Comorbidity Index; *BUC*: Bladder urine culture; *mPCNL*: mini percutaneous nephrolithotomy

*P value according to the Mann-Whitney U test for continuous data and the Fisher Exact Test for categorical variables, as indicated

Table 3 shows univariate (UVA) and multivariate (MVA) logistic regression analyses of factors associated with postoperative infectious complications. In the MVA, female gender (OR 2.1; *p* = 0.02), preoperative positive BUC (OR 3.6; *p* < 0.01), and residual stones (OR 0.21; *p* = 0.01) were identified as independent predictors of post-PCNL infection, after adjusting for age and stone volume.

**Table 3:** Logistic regression models predicting infectious complications in the whole cohort

	OR	P value	95% CI	OR	P value	95% CI
	UVA model			MVA model		
Age	1.1	0.3	0.97 – 1.25			
Female gender	2.3	<0.01	1.12 - 4.95	2.1	0.02	1.02 – 3.76
CCI≥1	1.1	0.57	0.72 - 1.78			
Stone Volume	1.1	<0.01	1.04 - 1.23	1.2	0.1	0.98 - 1.56
Stone density (HU)	0.9	0.2	0.98 - 1.09			
Preoperative indwelling stent	1.1	0.2	0.96 – 1.34			
Preoperative positive BUC	2.9	0.01	1.81 – 4.86	3.6	<0.01	1.82 - 9.45
Operative time	1.1	0.01	1.02 - 1.34	1.08	0.1	0.97 – 1.12
mPCNL vs standard	0.4	0.01	0.21 – 0.62			
Stone free status	0.3	0.01	0.23 – 0.61	0.21	0.01	0.09 – 0.52

*UVA *:Univariate model; *MVA*: Multivariate model, *CCI* :Charlson Comorbidity Index; *BUC:* Bladder urine culture; *mPCNL:* mini percutaneous nephrolithotomy;

Table 4 compares baseline and intraoperative parameters between patients with and without postoperative infectious complications, stratified by gender.

In males, patients with infections had a higher comorbidity burden (CCI ≥ 1, *p* = 0.01), larger stone volumes (*p* = 0.01), higher rates of positive BUC, and longer operative times (*p* = 0.01) than those without infections. SFR was lower in males with infections (76.6% vs. 84.3%, *p* = 0.01). In females, those with infections also had significantly larger stone volumes (*p* = 0.01), longer operative times (*p* = 0.01), and higher rates of positive BUC (*p* = 0.01) compared to females without infections. SFR was lower in females with infections (60.4% vs. 82.1%, *p* = 0.01). Notably, stone volume and operative time in females with infections were significantly lower than in males with infections [2.8 (1.3–15.5) vs. 5.5 (2.7–15.5) cm³, *p* = 0.01; 115 (95–150) vs. 130 (85–166) min, *p* = 0.01].

**Table 4:** Demographic characteristics and descriptive statistics of patients according to infections development in the male and female group

	Male cohort			Female cohort		
	Infections+ Infections		p-value^*^	Infections+ Infections		p-value^*^
No. of patients [No. (%)]	249 (83.3)	50 (16.7)		145 (75.1)	48 (24.9)	
Age (years)			0.7			0.2
Median (IQR)	59 (49-66)	58 (49-64)		59 (48-70)	56 (49-63)	
Range	20 - 85	20 – 82		24 – 81	24 - 81	
BMI (kg/m^2^)			0.6			0.7
Median (IQR)	24.8 (22.5-27.7)	24.9 (23.4-28.1)		24.6 (21.9-27.6)	25.1 (21.1-30.2)	
Range	17.9 – 36.8	18.1 – 36.6		18.1 – 38.8	18.5 – 38.8	
CCI			0.02			0.1
Mean (SD)	0.6 (0.9)	1.2 (1.4)		0.8 (1.3)	0.7 (0.9)	
Median (IQR)	0 (0-1)	0 (0-2)		0 (0-1)	0 (0-1)	
Range	0 – 5	0 – 5		0 – 6	0 - 5	
CCI ≥1 [No. (%)]	91 (36.5)	27 (54.0)	0.01	61 (42.1)	14 (29.2)	0.1
Stone volume (cm^3^)			0.01			0.01
Median (IQR)	2.6 (0.9-4.8)	5.5 (2.7-15.5)		1.9 (0.9-3.6)	2.8 (1.3-15.5)	
Range	0.5 – 61.2	1.2 – 53.5		0.5 – 18.9	0.5 – 48.8	
Multiple stones [No. (%)]	224 (74.9)	135 (69.9)	0.2	95 (65.5)	40 (83.3)	0.02
Staghorn stone [No. (%)]	62 (24.8)	29 (58.0)	0.01	49 (33.8)	22 (45.8)	0.1
Mean stone density (Hounsfield unit)			0.8			0.9
Median (IQR)	841 (585-1003)	858 (605-925)		900 (645-1032)	750 (560-940)	
Range	150 – 1983	450 – 1200		190 – 1500	300 - 1500	
Preoperative positive BUC No. [(%)]	29 (11.6)	19 (38.0)	0.01	35 (24.1)	19 (42.2)	0.01
mPCNL procedure [No. (%)]	216 (86.7)	32 (64.0)	0.01	129 (88.9)	39 (81.3)	0.2
Multiple access tracts [No. (%)]	34 (13.7)	13 (26.0)	0.02	16 (11.0)	15 (31.3)	0.01
Operative time (min)			0.01			0.01
Median (IQR)	90 (75-120)	130 (85-166)		90 (70-113)	115 (95-150)	
Range	40 – 180	50 – 180		30 – 180	48 - 180	
Hospitalization time (days)			0.01			0.01
Median (IQR)	4 (3-6)	9 (6-13)		4 (3-5)	7 (5-10)	
Range	2 – 50	3 – 40		2 – 18	3 - 42	
Infected stone [No. (%)]	31 (12.4)	21 (42.0)	0.001	40 (27.5)	23 (47.9)	0.01
Stone free rate [No. (%)]	210 (84.3)	30 (76.6)	0.01	119 (82.1)	29 (60.4)	0.01

*BMI:* body mass index; *CCI:* Charlson Comorbidity Index; *BUC:* Bladder urine culture; *mPCNL:* mini percutaneous nephrolithotomy

*p < 0.01 vs. +infections group of the male cohort.*

Table 5 presents gender-stratified logistic regression analyses of predictors for postoperative infectious complications. In males, MVA identified preoperative positive BUC (OR 1.8; *p* < 0.001) and stone-free status (OR 0.22; *p* = 0.02) as independent predictors of infection, after adjusting for CCI, stone volume, and operative time. In females, operative time (OR 1.1; *p* = 0.01), preoperative positive BUC (OR 2.0; *p* = 0.01), and SFR (OR 0.2; *p* = 0.02) were independently associated with postoperative infections, after accounting for stone volume.

**Table 5:** Logistic regression models predicting infectious complications in the male and female cohort

	UVA model		MVA model	
Male cohort	OR, p-value	95% CI	OR, p-value	95% CI
Age	1.1, 0.7	0.96 – 1.35		
CCI≥1	2.0, 0.02	1.09 - 3.79	2.1; 0.1	0.69 – 6.45
Stone Volume	1.1; <0.01	1.01 - 1.29	1.1; 0.2	0.97 - 1.21
Preoperative positive BUC	1.9; 0.01	1.11 – 4.86	1.8; <0.001	1.21 - 5.87
Operative time	1.1; 0.01	1.02 - 1.37	1.01; 0.4	0.98 – 1.45
mPCNL vs standard	0.2; 0.01	0.13 – 0.60		
Stone free status	0.4; 0.01	0.22 – 0.83	0.22; 0.02	0.06 – 0.83
Female cohort				
Age	1.1, 0.4	0.96 – 1.95		
CCI≥1	1.0, 0.6	0.82 - 1.23		
Stone Volume	1.2; <0.01	1.05 - 1.31	1.1; 0.5	0.91 - 1.21
Preoperative positive BUC	2.1; 0.01	1.02 – 6.62	2.0; 0.01	1.12 - 6.67
Operative time	1.1; 0.01	1.01 - 1.44	1.1; 0.01	1.02 – 1.93
mPCNL vs standard	0.5; 0.1	0.22 – 1.33		
Stone free status	0.3; 0.01	0.16 – 0.65	0.2; 0.02	0.03 – 0.48
Infected stone	1.0; 0.3	0.85 – 1.56		

*UVA*: Univariate model; *MVA:* Multivariate model, *CCI:* Charlson Comorbidity Index; *BUC:* Bladder urine culture; *mPCNL:* mini percutaneous nephrolithotomy

**Fig. 1:**
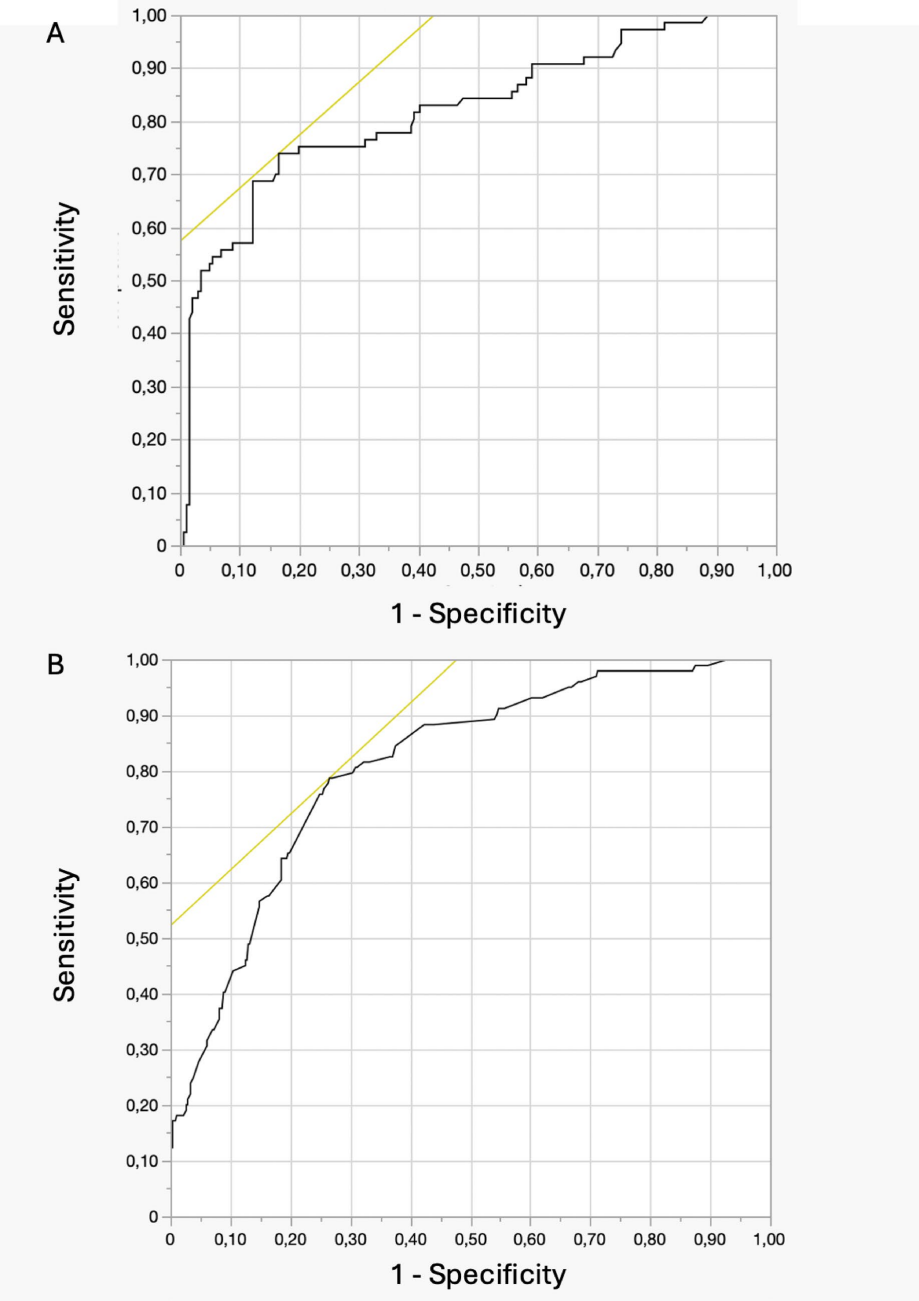
Receiver Operating Characteristic (ROC) curve analysis showing the predictive ability of stone volume (**A**) and operative time (**B**) for infectious complications in the female cohort

**Fig. 2:**
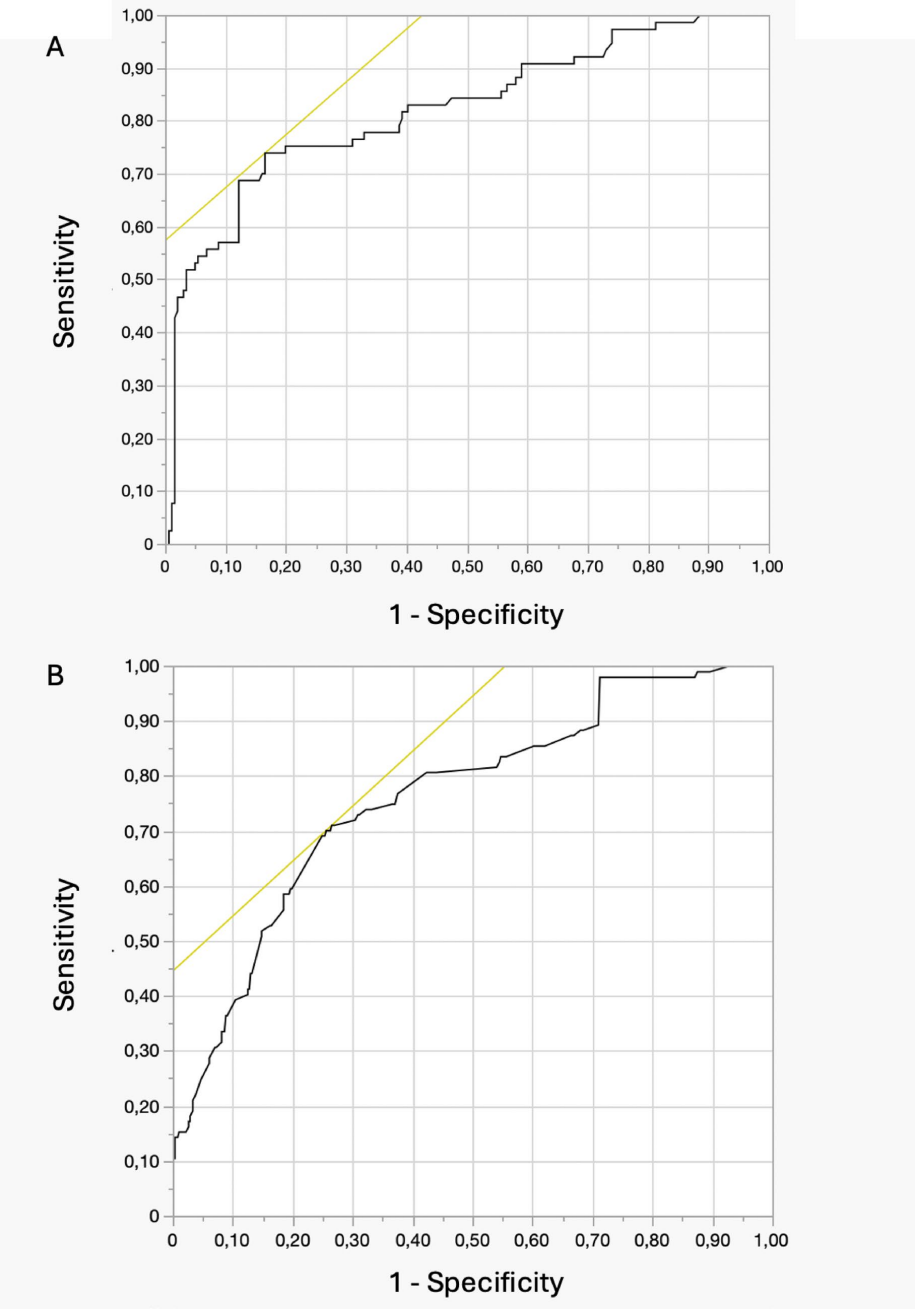
Receiver Operating Characteristic (ROC) curve analysis showing the predictive ability of stone volume (**A**) and operative time (**B**) for infectious complications in the male cohort

ROC curves showed that, in the female group, stone volume of 3.9 cm^3^ and operative time of 96 min had the best predictive ability for post-PCNL infectious complications. In men, stone volume of 5.1 cm^3^ and operative time of 137 min were the best predictors for infections (Table 6)(Figs. 1 and 2).

**Table 6:** ROC curves for predictors of infectious complications in the male and female cohort

	Youden index	Sensitivity	Specificity	AUC	Youden index	Sensitivity	Specificity	AUC
Male					Female			
Stone volume (cm^3^)	5.1	75.2%	73.1%	0.80	3.9	75.2%	72.9%	0.79
Operative time (min)	137	74.9%	72.8%	0.77	96	76.1%	75.6%	0.81

## Discussion

The primary aim of our study was to evaluate gender-specific predictors of infectious complications following PCNL in a large, real-world cohort of patients with kidney stones.

We showed that, in our cohort, females were at higher risk of post-PCNL infections than males. Preoperative positive BUC and residual stones were independent predictors in both sexes, while operative time had a greater impact in females. ROC analysis showed that females developed infections at lower stone volumes and shorter operative times than males, suggesting sex-specific differences in susceptibility to surgical stress. These findings provide a novel and detailed understanding of sex-specific risk factors for postoperative infections after PCNL, highlighting the importance of individualized risk stratification.

Across literature, several risk factors have been consistently associated with postoperative infectious complications in the general population. These include older age, larger stone and tract sizes, longer operative time, positive preoperative BUC, multiple access tracts, hydronephrosis, and struvite stone composition [6, 8, 16, 22, 23]. Our results corroborate these studies and confirm the higher risk of infections following standard PCNL than miniaturized procedures. Although standard PCNL is generally associated with lower irrigation pressure, it is typically reserved for larger and more complex stones, which likely explains the higher rate of postoperative infectious complications in this group. Conversely, mPCNL, despite potentially higher irrigation pressures, in our cohort, is usually performed using suction techniques that reduce intra-renal pressure (IRP) by continuously aspirating irrigation fluid and stone fragments. This not only shortens operative and lithotripsy times but also lower infection risk [18, 24, 25]. 

This study further emphasized the influence of gender-specific differences in terms of post-PCNL infectious complications. Xun et al. also reported a significant association between female gender, positive urine culture, and postoperative fever, supporting the hypothesis of a sex-related predisposition to infectious complications [26]. Although female sex has been frequently identified as a risk factor, to the best of our knowledge, no previous work has systematically investigated gender-stratified predictors of infectious complications following PCNL.

In our study, postoperative infections in males appeared to be primarily driven by preoperative infectious parameters, such as positive BUC, as well as the presence of residual stones, suggesting the persistence of infection-related risk factors and incomplete stone clearance may play a central role in the development of complications. Furthermore, residual fragments following PCNL have been shown to significantly influence patient outcomes [27]. In females, in addition to positive BUC and lower SFR, longer operative time emerged as the most relevant predictors of infectious events, highlighting the potential impact of surgical burden and procedural efficacy in this subgroup. Further supporting this sex-specific pattern, we showed that stone volume and operative time of females with infections were significantly lower than those of males who had infections complications, suggesting a potentially increased susceptibility or differing physiological response to surgical stress.

The higher susceptibility of female patients to post-PCNL infectious complications at lower stone volumes and shorter operative times may be explained by several biologically plausible mechanisms. Women have higher baseline rates of bacteriuria and urinary tract infections, in part due to anatomical differences, which facilitate bacterial ascent [28]. In patients with urolithiasis, the urinary microbial spectrum also differs by sex, with Escherichia coli being more frequently isolated in females [29]. Moreover, females are more likely to develop infection-related stones, such as struvite or carbonate apatite calculi, which are directly associated with bacterial colonization and may predispose to postoperative infectious events [30]. 

Interestingly, a similar host-related susceptibility has been observed in a large pediatric PCNL cohort, where prolonged operative time was an independent risk factor for postoperative SIRS [31]. Underweight children were particularly vulnerable to the inflammatory effects of longer procedures, supporting the notion that host biological characteristics can significantly influence the systemic inflammatory response to PCNL.

Recognizing that males and females may differ in their susceptibility and response to surgical stress highlights the need for personalized strategies that consider sex-specific risk profiles. Such an approach could optimize patient outcomes by guiding surgical planning, perioperative care, and postoperative monitoring. For example, in female patients, particular attention should be given to minimizing operative time; therefore, establishing operative time limits and utilizing instruments or techniques that expedite the procedure may be beneficial [18, 23, 24]. 

Our study has several strengths. It is innovative since it is the first to assess risk factors for infectious complications, stratified by gender, in a relatively large, homogeneous cohort of patients undergoing PCNL for kidney stones. Moreover, our results are relevant and with a strong clinical application, as they revealed that females are at higher risk of infectious complications than males. This study also highlights that commonly recognized predictors of infection in clinical practice should be interpreted with consideration of patient gender to more accurately assess the risk of infectious complications following PCNL. As a pilot study, it provides valuable preliminary evidence and establishes a foundation for future prospective cohort studies aimed at further exploring gender-specific differences in this context.

However, our study is not devoid of limitations. Larger multi-center studies are needed to externally validate our results. The retrospective nature of our study, spanning a 9-year period during which surgical techniques, instrumentation, and perioperative management evolved, may have influenced patient outcomes. Additional limitations include the single-center setting, which may affect the generalizability of the findings, and the lack of assessment of sex hormone levels, which could provide deeper insights into the biological mechanisms underlying gender differences in postoperative infection risk. Lastly, we lacked precise data about resistance pattern of each positive urine culture that could be of clinical relevance to increase the understanding of the post-op infectious cascade.

## Conclusion

Female patients were at higher risk of post-PCNL infections than males. Infections in females were mainly associated with preoperative BUC, operative time, and stone-free status, whereas in males, preoperative BUC and residual stones were key factors. Notably, females developed infections at lower stone volumes and shorter operative times, highlighting the need for a gender-tailored approach to optimize outcomes and reduce postoperative infections.

## Data Availability

No datasets were generated or analysed during the current study.

## References

[1] De Lorenzis E, Zanetti SP, Boeri L, Montanari E (2022) Is there still a place for percutaneous nephrolithotomy in current times? J Clin Med 11:5157. 10.3390/jcm11175157 36079083 10.3390/jcm11175157 PMC9457409

[2] Skolarikos A, Geraghty R, Somani B, Tailly T, Jung H, Neisius A et al (2025) European association of urology guidelines on the diagnosis and treatment of urolithiasis. Eur Urol 88:64–75. 10.1016/j.eururo.2025.03.011 40268592 10.1016/j.eururo.2025.03.011

[3] Zanetti SP, Boeri L, Gallioli A, Talso M, Montanari E (2017) Minimally invasive PCNL-MIP. Arch Esp Urol 70:226–234 28221157

[4] Zanetti SP, Talso M, Palmisano F, Longo F, Gallioli A, Fontana M et al (2018) Comparison among the available stone treatment techniques from the first European Association of Urology Section of Urolithiasis (EULIS) survey: do we have a queen? PLoS One 13:e0205159. 10.1371/journal.pone.0205159 30388123 10.1371/journal.pone.0205159 PMC6214503

[5] Grosso AA, Sessa F, Campi R, Viola L, Polverino P, Crisci A et al (2021) Intraoperative and postoperative surgical complications after ureteroscopy, retrograde intrarenal surgery, and percutaneous nephrolithotomy: a systematic review. Minerva Urol Nephrol. 10.23736/S2724-6051.21.04294-4 33887891 10.23736/S2724-6051.21.04294-4

[6] Yu Y, Pu J, Wu T, Hu L (2021) The characteristics and influencing factors of fever in postoperative patients undergoing percutaneous nephrolithotomy: a retrospective analysis. Medicine (Baltimore) 100:e26485. 10.1097/MD.0000000000026485 34397870 10.1097/MD.0000000000026485 PMC8360468

[7] Talmor M (1999) Relationship of systemic inflammatory response syndrome to organ dysfunction, length of stay, and mortality in critical surgical illness: effect of intensive care unit resuscitation. Arch Surg 134:81. 10.1001/archsurg.134.1.81 9927137 10.1001/archsurg.134.1.81

[8] Lai WS, Assimos D (2018) Factors associated with postoperative infection after percutaneous nephrolithotomy. Rev Urol 20:7–11. 10.3909/riu0778 29942195 10.3909/riu0778 PMC6003297

[9] Rashid AO, Fakhulddin SS (2016) Risk factors for fever and sepsis after percutaneous nephrolithotomy. Asian J Urol 3:82–87. 10.1016/j.ajur.2016.03.001 29264169 10.1016/j.ajur.2016.03.001 PMC5730806

[10] Cao JD, Wang ZC, Wang YL, Li HC, Gu CM, Bai ZG et al (2022) Risk factors for progression of urolith associated with obstructive urosepsis to severe sepsis or septic shock. BMC Urol 22:46. 10.1186/s12894-022-00988-8 35346141 10.1186/s12894-022-00988-8 PMC8962082

[11] Chugh S, Pietropaolo A, Montanari E, Sarica K, Somani BK (2020) Predictors of urinary infections and urosepsis after ureteroscopy for stone disease: a systematic review from EAU section of urolithiasis (EULIS). Curr Urol Rep 21:16. 10.1007/s11934-020-0969-2 32211969 10.1007/s11934-020-0969-2

[12] Charlson ME, Pompei P, Ales KL, MacKenzie CR (1987) A new method of classifying prognostic comorbidity in longitudinal studies: development and validation. J Chronic Dis 40:373–383. 10.1016/0021-9681(87)90171-8 3558716 10.1016/0021-9681(87)90171-8

[13] Ito H, Kawahara T, Terao H, Ogawa T, Yao M, Kubota Y et al (2013) Evaluation of preoperative measurement of stone surface area as a predictor of stone-free status after combined ureteroscopy with Holmium laser lithotripsy: a single-center experience. J Endourol 27:715–21. 10.1089/end.2012.0548 23402348 10.1089/end.2012.0548

[14] Ripa F, De Lorenzis E, Passarelli F, Zanetti SP, Boeri L, Albo G et al (2025) Urine culture in endourology - clinical relevance, strengths and controversies. Curr Urol Rep 26:80. 10.1007/s11934-025-01312-1 41396226 10.1007/s11934-025-01312-1

[15] De Lorenzis E, Boeri L, Gallioli A, Fontana M, Zanetti SP, Longo F et al (2021) Feasibility and relevance of urine culture during stone fragmentation in patients undergoing percutaneous nephrolithotomy and retrograde intrarenal surgery: a prospective study. World J Urol 39:1725–32. 10.1007/s00345-020-03387-6 32734462 10.1007/s00345-020-03387-6 PMC8217000

[16] Silvani C, Zanetti SP, Boeri L, Turetti M, Matinato C, Teri A et al (2023) The clinical role of bacteremia and bacterial spread into the irrigation fluid during percutaneous nephrolithotomy: a prospective study. World J Urol 41:135–42. 10.1007/s00345-022-04217-7 36469113 10.1007/s00345-022-04217-7

[17] Zanetti SP, Lievore E, Fontana M, Turetti M, Gallioli A, Longo F et al (2021) Vacuum-assisted mini-percutaneous nephrolithotomy: a new perspective in fragments clearance and intrarenal pressure control. World J Urol 39:1717–23. 10.1007/s00345-020-03318-5 32591902 10.1007/s00345-020-03318-5 PMC8217021

[18] Lievore E, Boeri L, Zanetti SP, Fulgheri I, Fontana M, Turetti M et al (2021) Clinical comparison of mini-percutaneous nephrolithotomy with vacuum cleaner effect or with a vacuum-assisted access sheath: a single-center experience. J Endourol 35:601–8. 10.1089/end.2020.0555 33076705 10.1089/end.2020.0555

[19] Boeri L, Turetti M, Silvani C, Fulgheri I, Jannello LMI, Garbagnati S et al (2022) The comprehensive complication index as a tool for reporting the burden of complications after mini-percutaneous nephrolithotomy: is it time to leave the Clavien–Dindo classification behind? World J Urol 40:1829–37. 10.1007/s00345-022-04045-9 35643945 10.1007/s00345-022-04045-9 PMC9236985

[20] Xu P, Zhang S, Zhang Y, Zeng T, Chen D, Wu W et al (2022) Preoperative antibiotic therapy exceeding 7 days can minimize infectious complications after percutaneous nephrolithotomy in patients with positive urine culture. World J Urol 40:193–9. 10.1007/s00345-021-03834-y 34550426 10.1007/s00345-021-03834-y

[21] Jannello LMI, Turetti M, Silvani C, Galbiati G, Garbagnati S, Pozzi E et al (2022) Urologists are optimistic surgeons: prevalence and predictors of discordance between intraoperative stone-free rate and cross-sectional imaging evaluation after vacuum-assisted mini-percutaneous nephrolithotomy. World J Urol 40:2331–8. 10.1007/s00345-022-04091-3 35831471 10.1007/s00345-022-04091-3 PMC9427905

[22] Pozzi E, Malfatto M, Turetti M, Silvani C, Jannello LMI, Garbagnati S et al (2022) Validation of the Trifecta Scoring Metric in Vacuum-Assisted Mini-Percutaneous Nephrolithotomy: a single-center experience. J Clin Med 11:6788. 10.3390/jcm11226788 36431265 10.3390/jcm11226788 PMC9697932

[23] Lievore E, Zanetti SP, Fulgheri I, Turetti M, Silvani C, Bebi C et al (2022) Cost analysis between mini-percutaneous nephrolithotomy with and without vacuum-assisted access sheath. World J Urol 40:201–11. 10.1007/s00345-021-03811-5 34432135 10.1007/s00345-021-03811-5 PMC8813798

[24] Marmiroli A, Nizzardo M, Zanetti SP, Lucignani G, Turetti M, Silvani C et al (2024) Vacuum-assisted mini-percutaneous nephrolithotomy is associated with lower rates of infectious complications compared to vacuum-cleaner procedure in patients at high risk for infections: a single-center experience. World J Urol 42:200. 10.1007/s00345-024-04897-3 38536503 10.1007/s00345-024-04897-3 PMC10973077

[25] Nizzardo M, Li Puma A, Graps G, Ciamarra F, Lucignani G, Parolin V et al (2025) Vacuum-assisted mini-percutaneous nephrolithotomy is associated with lower rates of infectious complications compared to standard procedures in low-risk patients: a single-center experience. World J Urol 43:457. 10.1007/s00345-025-05783-2 40711573 10.1007/s00345-025-05783-2

[26] Xun Y, Yang Y, Yu X, Li C, Lu J, Wang S (2020) A preoperative nomogram for sepsis in percutaneous nephrolithotomy treating solitary, unilateral and proximal ureteral stones. PeerJ 8:e9435. 10.7717/peerj.9435 32655994 10.7717/peerj.9435 PMC7331651

[27] Li Puma A, Passarelli F, De Lorenzis E, Montanari E, Albo G, Boeri L (2025) Residual fragments after percutaneous nephrolithotomy: is it mandatory to treat them all? Urolithiasis 53:107. 10.1007/s00240-025-01775-4 40465007 10.1007/s00240-025-01775-4

[28] Deltourbe L, Lacerda Mariano L, Hreha TN, Hunstad DA, Ingersoll MA (2022) The impact of biological sex on diseases of the urinary tract. Mucosal Immunol 15:857–866. 10.1038/s41385-022-00549-0 35869147 10.1038/s41385-022-00549-0 PMC9305688

[29] Gu J, Chen X, Yang Z, Bai Y, Zhang X (2022) Gender differences in the microbial spectrum and antibiotic sensitivity of uropathogens isolated from patients with urinary stones. J Clin Lab Anal 36:e24155. 10.1002/jcla.24155 34854120 10.1002/jcla.24155 PMC8761408

[30] Chien T-M, Lu Y-M, Li C-C, Wu W-J, Chang H-W, Chou Y-H (2021) A retrospective study on sex difference in patients with urolithiasis: who is more vulnerable to chronic kidney disease? Biol Sex Differ 12:40. 10.1186/s13293-021-00382-3 34099045 10.1186/s13293-021-00382-3 PMC8185917

[31] Abudurexiti N, Li X, Liu B, Wang S, Dong Q (2025) Saturation effect between operation time and post-PCNL SIRS in pediatric patients. World J Urol 43:498. 10.1007/s00345-025-05869-x 40824490 10.1007/s00345-025-05869-x

